# The influence of malalignment and ageing following sterilisation by gamma irradiation in an inert atmosphere on the wear of ultra-high-molecular-weight polyethylene in patellofemoral replacements

**DOI:** 10.1177/0954411917696112

**Published:** 2017-03-25

**Authors:** Raman Maiti, Raelene M Cowie, John Fisher, Louise M Jennings

**Affiliations:** Institute of Medical and Biological Engineering, University of Leeds, Leeds, UK

**Keywords:** Knee prostheses, patellofemoral joint, wear, arthroplasty, in vitro

## Abstract

Complications of patellofemoral arthroplasty often occur soon after implantation and, as well as other factors, can be due to the design of the implant or its surgical positioning. A number of studies have previously considered the wear of ultra-high-molecular-weight polyethylene patellae following suboptimal implantation; however, studies have primarily been carried out under a limited number of degrees of freedom. The aim of this study was to develop a protocol to assess the wear of patellae under a malaligned condition in a six-axis patellofemoral joint simulator. The malalignment protocol hindered the tracking of the patella centrally in the trochlear groove and imparted a constant 5° external rotation (tilt) on the patella button. Following 3 million cycles of wear simulation, this condition had no influence on the wear of ultra-high-molecular-weight polyethylene patellae aged for 4 years compared to well-positioned non-aged implants (p > 0.05). However, under the malaligned condition, ultra-high-molecular-weight polyethylene patellae aged 8–10 years after unpacking (following sterilisation by gamma irradiation in an inert atmosphere) and worn ultra-high-molecular-weight polyethylene components also aged 4 years after unpacking (following the same sterilisation process) exhibited a high rate of wear. Fatigue failure due to elevated contact stress led to delamination of the ultra-high-molecular-weight polyethylene and in some cases complete failure of the patellae. The results suggest that suboptimal tracking of the patella in the trochlear groove and tilt of the patella button could have a significant effect on the wear of ultra-high-molecular-weight polyethylene and could lead to implant failure.

## Introduction

Through the 1970s (35%) and 1980s (68%), there was an increase in the popularity of replacing the patella during total knee replacement (TKR).^1^ However, there is still debate as to whether the patella should be resurfaced routinely^2^ during TKR or whether a more selective approach should be taken.^3^ While there have been shown to be functional benefits of resurfacing the patella,^4^ and a reduction in the reoperation rate due to patellofemoral joint (PFJ) problems,^5^ failure of the patella button can occur due to loosening, fracture, infection, instability, mal-tracking, wear and overstuffing.^3,6–8^

Along with the patella resurfacing carried out during TKR, approximately 10,000 unicompartmental PFJ replacements are carried out in England and Wales annually.^6^ These implants are often used as a conservative approach in younger patients. The National Joint Registry (NJR) reports a relatively high revision rate of unicompartmental PFJ implants compared to hip and knee prostheses, approximately 20% at 10 years with failure due to pain, progressive arthritis of the tibiofemoral joint and loosening often resulting in revision of the joint to a TKR.^6,9^ However, when comorbidities such as degeneration of the tibiofemoral joint do not occur, a rate of survivorship >90% at 17 years has been reported for unicompartmental PFJ implants.^10^

There have been several experimental studies investigating the wear of the PFJ. However, some studies were carried out under a limited number of degrees of freedom,^11,12^ and constraint of some motions may protect the system from the effects of malalignment. Simulators and test protocols with an increased number of degrees of freedom more representative of the in vivo scenario have also been developed.^13–15^ Under a standard gait cycle, reported wear rates of ultra-high-molecular-weight polyethylene (UHMWPE) patellae (which had not been aged and were well aligned) have ranged from 0.3 mm^3^/million cycles (MC)^14^ through 3.1^13^ to 6.3 mm^3^/MC^15^ in six-axis simulation, with a bow-tie-shaped wear scar typical of those seen on retrieved implants.^16^

As joint arthroplasty is increasingly being used in younger, more active patients who place greater demands on their implants, there is a need to develop enhanced pre-clinical test methods to understand how variations in surgical delivery influence wear. For the hip and tibiofemoral joint, protocols have been developed to study conditions such as edge loading due to variations in rotational and translational positioning,^17^ and femoral condylar lift-off, respectively.^18^ The failure mechanisms of PFJ replacements show that component design and positioning play a role in the longevity of the implant with failure often occurring soon after implantation (<2 years).^19^ The position of the patella button on the patella including the depth and angle of resected bone can influence its tracking, stability and tilt^20–22^ which can influence the wear of the implant.^14^ The wear rate of the patella button must be as low as possible as its debris contributes to the total volume of wear debris from a TKR,^23^ which should be minimised to reduce the potential for implant failure due to wear debris-induced osteolysis.^24^ Therefore, there is a need to test implants under more rigorous conditions.^25^

Several protocols have been developed for wear simulation of patellae under enhanced test conditions. Under stair-climbing kinematics that apply a high compressive load and large flexion–extension of the femoral component, subsurface cracking has been observed on aged conventional UHMWPE caused by high contact stresses.^12^ Fixing the medial–lateral translation of the patella button to constrain its tracking in the trochlear groove has also been shown to increase wear under both standard gait and stair-climbing kinematics.^26^ Imparting up to 5° of internal rotation to the femoral component has been shown to lateralise the wear scar increasing the wear rate of new implants^14^ and causing cracking and delamination of aged UHMWPE under stair-climbing kinematics.^12^

As patient demand from their orthopaedic implants increases, there is a need for UHMWPE to retain its properties over a longer duration of implantation. One of the limitations of pre-clinical experimental wear simulation is the relatively short duration of the tests. A simulation of 1 MC is typically taken as being equivalent to 1 year in vivo;^27^ this takes approximately 12 days in the simulator, and the test duration does not reflect the age of the implant or potential in vivo oxidation.^28^ Therefore, to determine the implications of long-term use, accelerated ageing protocols such as ASTM F2003^29^ have been developed to replicate the oxidation of UHMWPE and to assess whether long-term implantation (>10 years) influences wear rate or mechanical properties of the UHMWPE.^28^ However, these ageing protocols do not necessarily reflect in vivo oxidation;^30^ therefore, in this study, a real-time ageing process in air was used to more closely replicate in vivo oxidation.

The GUR1020 UHMWPE patella buttons used in this study were gamma sterilised with 2.5–4 MRad in an inert atmosphere (vacuum) and barrier packaged in foil pouches (GVF: Gamma Vacuum Foil) to minimise oxidation during long-term shelf storage.^31^ Gamma sterilisation remains one of the most common sterilisation techniques due to cross-linking of the UHMWPE during the sterilisation process, which improves the mechanical properties and tribological performance of the UHMWPE compared to sterilisation of UHMWPE by gas plasma or ethylene oxide.^32^ Historically, gamma sterilisation was carried out in air; however, prolonged shelf storage and implantation both led to post-irradiation oxidative degradation of the UHMWPE which upon implantation delaminated leading to premature failure. This sterilisation method was discontinued in the mid- to late 1990s and current gamma irradiation techniques of sterilising UHMWPE in an inert atmosphere and barrier packaging means that oxidative degradation due to shelf ageing is now largely a historical problem.^31^ However, a by-product of the gamma sterilisation technique is the presence of microradicals in the polymer which remain irrespective of the sterilisation environment and there is a concern that these microradicals have the potential to cause UHMWPE oxidation.^33,34^

The aim of this study was to develop a methodology for experimental wear simulation of the patellofemoral joint under malaligned conditions and to determine the influence of malalignment on the wear performance of unworn and worn UHMWPE patellae aged either 4 or 8–10 years. It was hypothesised that ageing in air for 4 years has no influence on the wear performance of UHMWPE patellae.

## Materials and methods

The implants used were right, mid-size Press Fit Condylar (PFC) Sigma (DePuy Synthes Joint Reconstruction, Wasaw, Inc., USA) components. This is a commercially available and commonly used implant in the UK.^6^ CoCrMo femoral components were tested against 38 mm GUR1020 UHMWPE dome-shaped patella buttons which had been sterilised in foil pouches by gamma irradiation (2.5–4 Mrad) in a vacuum (GVF). The buttons were a combination of round and oval dome geometries and were divided into three groups as detailed in Table 1. The implants were either unworn or had previously undergone experimental wear simulation for 9 MC under well-positioned conditions (worn) and all the implants had been aged to varying degrees. The ageing process involved removal of the UHMWPE patellae from its barrier packaging and storing in air for up to 10 years prior to wear simulation; this protocol gave a real-time ageing process considered to be more representative of in vivo oxidation than accelerated ageing protocols. An additional two patella buttons were used as unloaded soak controls to compensate for the uptake of moisture by the implants.

**Table 1.** Patella buttons used in this study.

Geometry	Group 1 – unworn, aged 4 years		Group 2 – worn, aged 4 years		Group 3 – unworn, aged 8–10 years
	Round	Oval	Round	Oval	Round
Number of samples	1	2	3	3	3
Time removed from packing before testing (years)	4	4	4	4	8–10
Previous testing	No	No	9 MC well-positioned conditions^15^	9 MC well-positioned conditions^15^	No

**Figure 1.**
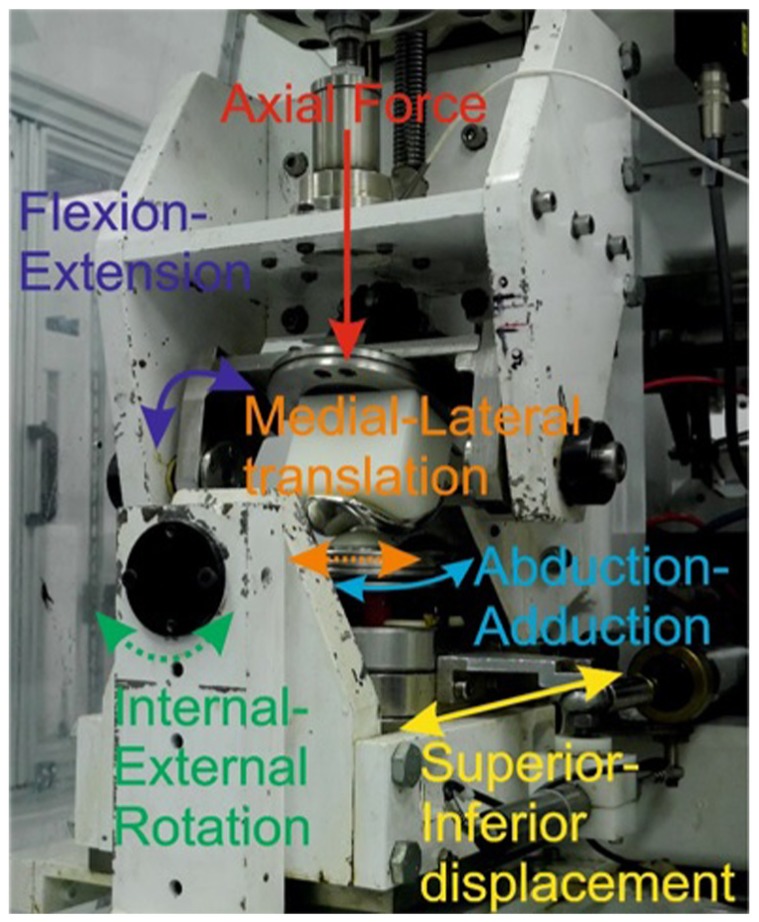
One station of the patellofemoral joint simulator showing the controlled (solid lines) and uncontrolled (dashed lines) axes of motion.

**Figure 2.**
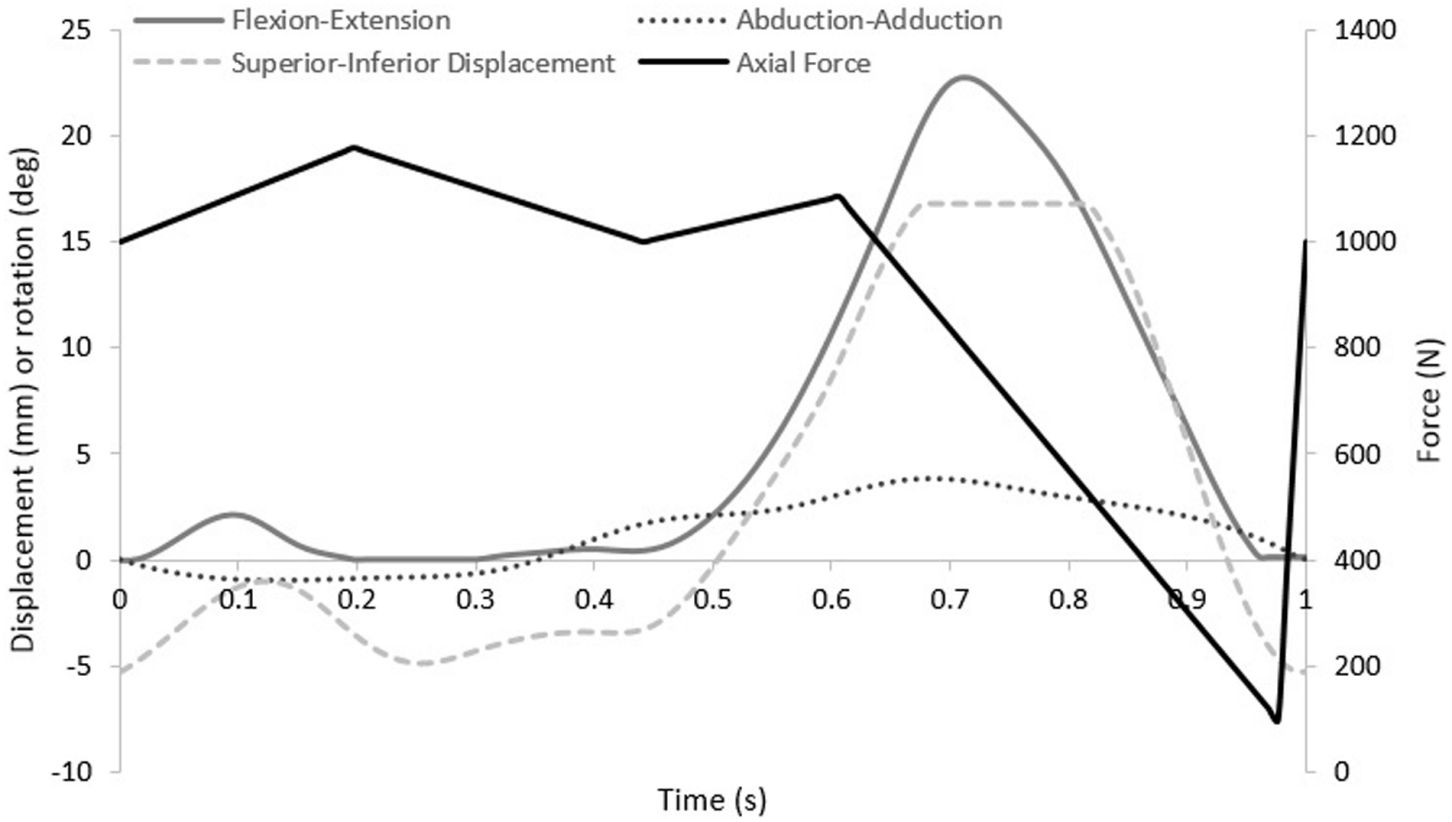
Simulator input kinematics.

Experimental wear simulation was carried out using a ProSim 6 station electropneumatic knee simulator (Simulation Solutions Ltd, Stockport, UK) modified for testing the PFJ. The simulator used has four controlled axes of motion and two passive axes. The controlled axes were flexion-extension (FE) of the femoral component, axial force (AF), superior–inferior (SI) translation and abduction–adduction (AA) rotation. The SI and AA were driven through the patella (Figure 1). The internal–external (IE) rotation and the medial–lateral displacement of the patella were free to move. The FE of the femoral component was driven through a range of 22°, SI translation was 5 to −17 mm, AA rotation was 1 mm representative of a low kinematic condition and the maximum AF was 1177 N. The input kinematics are shown in Figure 2 and have been detailed in previous work by Maiti et al.^15^

**Figure 3.**
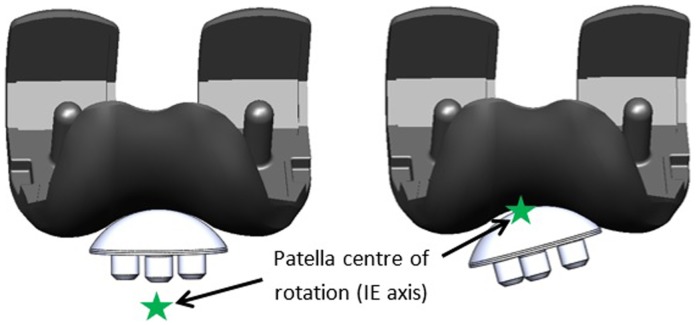
Left: well-positioned patella button. Right: malalignment of the patella by alteration of the centre of rotation of the patella in the IE axis to the surface of the component.

To create the malaligned condition, the centre of rotation of the patella button in the IE axis was moved from a point below the button to the articulating surface of the patella (Figure 3) inducing patella tilt (IE rotation). Patella tilt was measured using a potentiometer with readings averaged over 3 cycles every 0.3 MC.

The lubricant used was 25% bovine serum diluted with 0.03% (v/v) sodium azide solution to retard bacterial growth. The lubricant was replaced every 0.3 MC, and the tests were carried out for 3 MC or until failure of the patella button occurred.

The wear of the patella buttons was determined by their loss in mass measured by gravimetric analysis using a Mettler Toledo AT201 digital microbalance (Mettler Toledo, Ohio, USA). The mean surface roughness (Ra) of the articulating surfaces was assessed by contacting profilometry using a Taylor Hobson PGI 800 contacting form Talysurf (Taylor Hobson, Leicester, UK). A Gaussian filter and an upper cut-off of 0.8 mm were used for the polyethylene patellae.^35^

The mean area of the wear scar on the patellae was assessed as a percentage of the total area of the component by tracing around the wear scar, photographing the implant and analysing using ImageJ.^36^

The mean wear rates and surface roughness were calculated and expressed with ± 95% confidence limits. Statistical analysis was carried out using analysis of variance (ANOVA) with post hoc Tukey’s test in MiniTab 17^37^ with significance taken at p < 0.05.

The data associated with this article are openly available from the University of Leeds Data Repository.^38^

## Results

**Figure 4.**
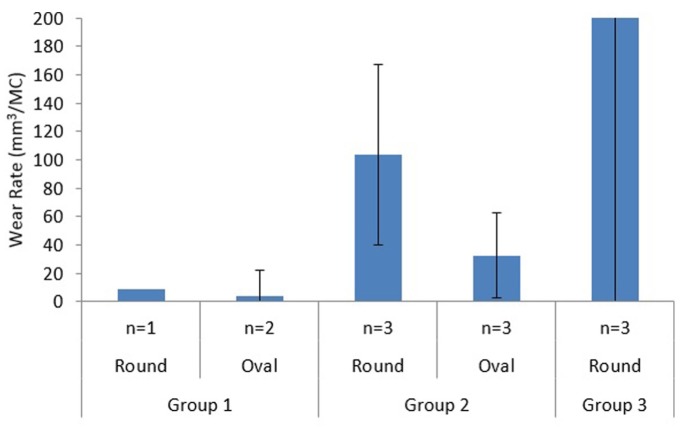
Mean wear rate of Group 1, Group 2 and Group 3 round and oval patella buttons (mm^3^/MC) under malalignment conditions. Mean wear rate for Group 3 implants was >2000 mm^3^/MC.

**Figure 5.**
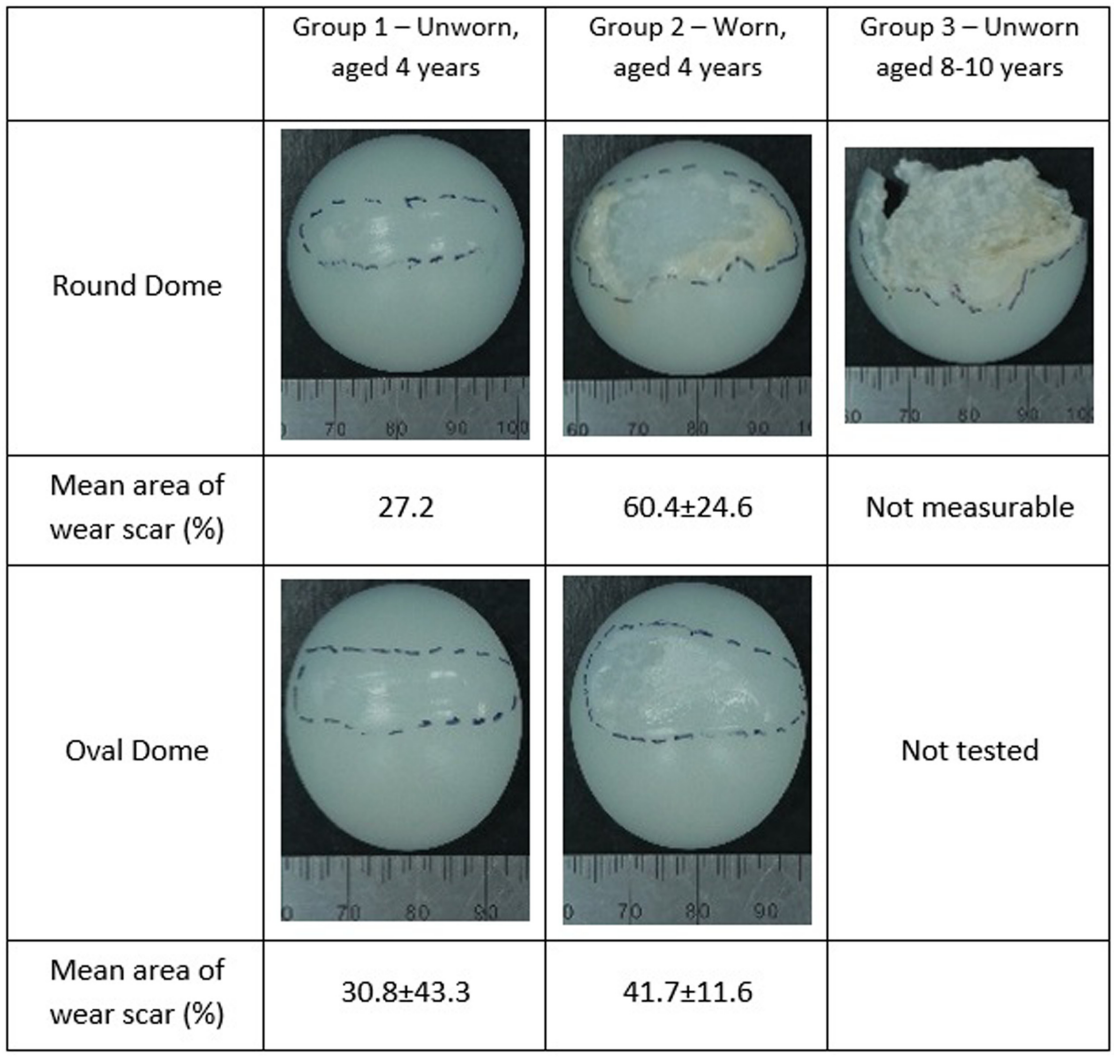
Images representative of the patella buttons after wear simulation under malaligned conditions; the wear scar is shown by the dashed lines and mean area of wear scar expressed as a percentage of the whole patella ± 95% confidence limits.

**Figure 6.**
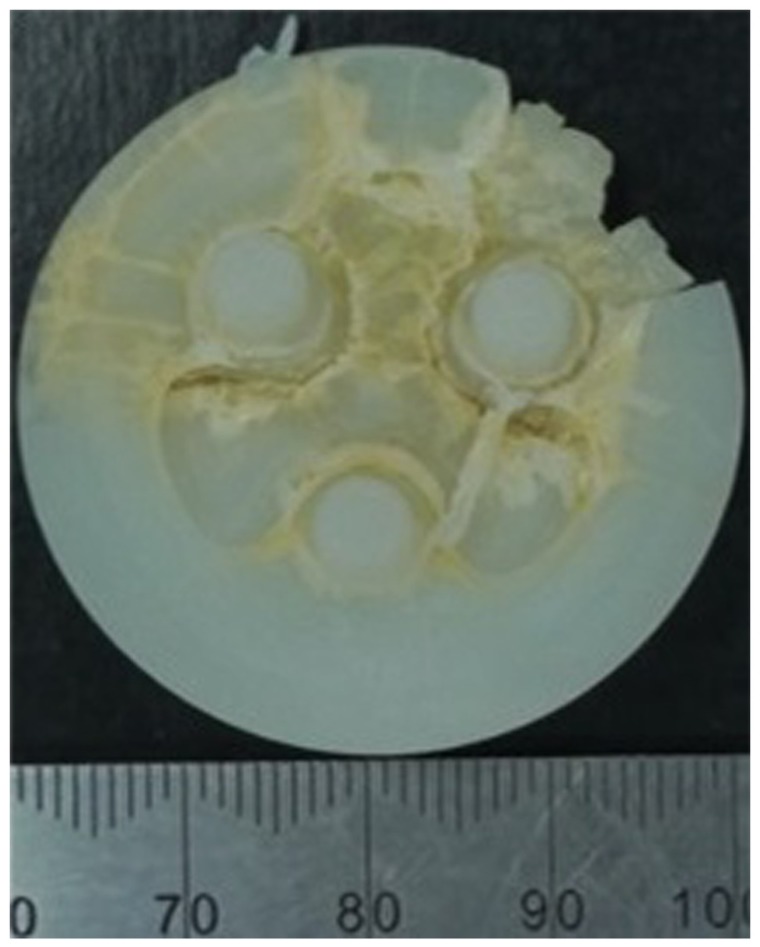
Patella from a Group 3 implant (aged 8–10 years) showing cracks propagating through the UHMWPE following wear simulation.

In this simulation model, moving the centre of rotation of the patella button in the IE axis from a position where the patella would be considered to be well positioned to the articulating surface of the implant resulted in a constant 5° external rotation of the patella button in all the samples throughout the gait cycle as shown in Figure 3. Table 2 shows the wear rates of the individual UHMWPE patella buttons from Groups 1 and 2 following experimental wear simulation. The mean wear rates of the unworn, aged 4 years patella buttons (Group 1) were 8.5 mm^3^/MC and 3.6 ± 18.2 mm^3^/MC for the round dome and oval dome implants, respectively (Figure 4). The implants that had previously been tested (for 9 MC) under well-positioned conditions (Group 2) had higher rates of wear, 103.7 ± 63.5 mm^3^/MC and 32.6 ± 29.8 mm^3^/MC for the round dome and oval dome implants, respectively (Figure 4). There was a significant difference (p = 0.001) in the wear rate of both the round and oval worn patella buttons (Group 2) compared to the unworn, aged 4 years implants (Group 1). There was evidence of subsurface cracking on all the Group 2 UHMWPE patella buttons; this was more prominent in the round dome implants where there was delamination of the UHMWPE (Figure 5). The implants aged in air for 10 years (Group 3) demonstrated gross failure with cracks propagating through the UHMWPE (Figures 5 and 6). For two of the Group 3 implants, the test was stopped after 1.5 MC; the mean wear rate of the patella buttons could not be accurately measured and was in excess of 2000 mm^3^/MC, and hence they have been excluded from Table 2 and the statistical analysis.

**Table 2.** Wear rates (mm^3^/MC) of individual patella buttons following wear simulation.

Implant	Group 1 – unworn, aged 4 years		Group 2 – worn, aged 4 years	
	Round	Oval	Round	Oval
1	8.5	2.2	120.7	21.1
2		5.0	74.3	45.1
3			116.2	31.7

Figure 5 shows the mean percentage area of the wear scar, which was larger in the Group 2 implants than the Group 1 implants for both round and oval dome patellae. It was not possible to assess the size of the wear scar on the Group 3 implants due to their failure and the shortened duration of the study.

Surface roughness measurements of the patella buttons were taken prior to and post wear simulation as shown in Table 3. The pre-test surface measurements were not the same for all the samples as the Group 1 and Group 3 UHMWPE implants were unworn and Group 2 UHMWPE implants had been previously tested. In the wear scar, polishing/burnishing was apparent, with some evidence of pitting and scratching. However, fatigue failure leading to delamination of the UHMWPE was the dominant wear mechanism, which led to an increase in the mean surface roughness of the implants. The femoral components had visible linear scratching in the femoral groove orientated in an SI direction.

**Table 3.** Mean surface roughness (Ra) with 95% confidence limits of the patella buttons taken prior to and post wear simulation.

Samples	Pre-test roughness (μm)	Post-test roughness (μm)
Group 1	0.92 ± 0.17	5.05 ± 11.17
Group 2	2.46 ± 0.96	11.48 ± 4.09
Group 3	1.24 ± 0.09	14.23 ± 16.48

## Discussion

As the use of implants increases in younger, more active patients with higher expectations from their joint replacement, it is important to understand how parameters such as patella tilt and tracking could influence the wear and longevity of an implant. The aim of this study was to develop a protocol for experimental wear simulation of patella buttons under malpositioned conditions.

This novel method for experimental wear simulation of the PFJ under malaligned conditions was developed by moving the centre of rotation in the IE axis to the surface of the patella button, imparting a 5° external rotation on the sample throughout the gait cycle. When patella tilt occurs in vivo, the degree of tilt varies with the flexion angle of the knee,^39^ and the constant tilt of the patella in this study created a worst case test by continually applying a high contact stress within a small contact area on the patellae. The constant external rotation shifted the position of the wear scar visible on the patellae from the previously reported symmetrical bow-tie-shaped wear scar^16^ to a more medially positioned wear scar where the patella came into contact with the edge of the femoral groove. A loss of symmetry in the wear scar on the patella button has also been observed by Vanbiervliet et al.^14^ who created an adverse wear test condition by internally rotating the femoral component. In an optimally positioned implant, the patella follows the profile of the trochlear groove and tracks centrally in the groove. However, this is only seen in approximately 55% of paients,^40^ so, suboptimal tracking and patella tilt are common. Rotation of the femoral component, tissue tension around the implant or the depth and angle of the resected bone can cause malalignment of the patella^19^ highlighting the need for the development of enhanced test protocols for the patella.

For the unworn, aged 4 years patellae (Group 1), malalignment had no influence on wear performance compared to well-positioned implants which could freely tilt and translate medially and laterally to follow the trochlear groove with mean wear rates of 3.6–8.5 mm^3^/MC for the malaligned components and 6.5–8.6 mm^3^/MC for well-positioned implants.^15^ Consistent with the findings of a previous study, when tested under malaligned conditions, the geometry of the patella button seemed not to influence its wear;^15^ however, in this study, the sample sizes of both geometries of patellae were small, so the results were not conclusive. Following 3 MC testing of unworn patellae under the malaligned condition, there was some evidence of subsurface cracking. This was thought to be caused by fatigue failure due to the higher contact stress caused by the patella tilt and contact with the edge of the trochlear groove. Hence, had the wear test been carried out for a longer duration, there was the potential for the UHMWPE to begin to exhibit delamination which could dramatically increase the wear rate.

The patella buttons which had previously been tested for 9 MC under well-positioned test conditions (Group 2) had a significantly higher rate of wear (p = 0.001) compared to the unworn patella buttons (Group 1) due to delamination of the UHMWPE. The wear mechanism was multifactorial and was attributed to a combination of the extended duration of testing (in excess of 12 MC in total) and the patellar tilt causing high contact stress in the malaligned condition. For the Group 2 implants, the mean wear rate was higher with the round patellae than the oval implants; however, a high variation was measured in the wear rates for both geometries of implant and there was no statistical significance (p > 0.05) in wear rate between the geometries of the implants. Again, the small sample size and the high variability in the wear meant that firm conclusions could not be drawn.

The Group 3 implants exhibited gross failure. Severe UHMWPE wear of gamma-sterilised patellae has been seen in retrieval studies and linked to the use of metal backings which give thinner UHMWPE bearings, which are more likely to fail as a result of cracking and delamination.^41^ However, this study used all UHMWPE patellae and considered the influence of ageing in air of UHMWPE gamma sterilised in an inert atmosphere for both 4 and 8–10 years. Following ageing in air for 4 years (Group 1), the wear rate of the UHMWPE was low and similar to the wear rates of well-positioned components,^15^ so 4 years of ageing in air had no influence on wear of irradiated UHMWPE over a 3 MC test. The Group 3 implants were aged for 8–10 years. Under malaligned test conditions, these patella buttons exhibited gross failure due to delamination, and for two of the three implants, the test was stopped before reaching 3 MC. The wear of these implants could not be determined accurately due to their failure; however, the visible delamination alluded to the high wear being as a result of oxidation. Delamination of patellae has previously been observed in wear tests carried out under aged and adverse conditions. For UHMWPE patellae gamma sterilised in oxygen, Burroughs et al. demonstrated subsurface cracking of artificially aged patella buttons when tested under malalignment and a stair-climbing gait cycle. The test protocol used reduced the contact area between the patella and femoral component which resulted in an increase in the contact pressure leading to delamination of the polyethylene.^12^ In this study, the ageing of UHMWPE in the Group 3 implants exceeded that of Burroughs et al.^12^ and the test conditions also resulted in a high contact stress condition, so it is likely that the failure of the patellae in this study occurred by a similar mechanism of delamination which was accelerated by oxidation of the polyethylene.

The sterilisation method of UHMWPE has a strong influence on its fatigue life and propensity for delamination. Historically, following shelf ageing, UHMWPE gamma sterilised (in air) showed delamination due to fatigue failure.^42^ Following gamma sterilisation, free radicals are present in the UHMWPE which when combined with oxygen lead to oxidative degradation of the UHMWPE. Historical gamma sterilisation in air led to significant oxidation of the UHMWPE during the sterilisation and packaging processes prior to implantation. These issues have been largely overcome by gamma sterilisation of UHMWPE in an inert atmosphere and barrier packaging. However, unless the UHMWPE is stabilised with an antioxidant such as Vitamin E,^43^ the free radicals, which are a by-product of the irradiation sterilisation process, still remain in the material irrespective of the sterilisation environment. Hence, there is the potential for these to react with oxygen in body fluids and oxidative degradation of the UHMWPE to occur in vivo which may reduce the lifespan of the implant.^33^ Delamination failure occurs due to a combination of oxidation of the UHMWPE and cyclic loading. Studies have shown that the oxidation index threshold for UHMWPE that has been gamma stabilised in an inert atmosphere is reached after ∼11–14 years in vivo due to a combination of the oxygen-rich environment and the cyclic loading of the UHMWPE.^33^ In this study, the Group 3 implants had a combination of long-term (8–10 years) ageing in air and the high contact stress of the malaligned components replicated the delamination failure mode understood to be driven by the oxidation of the UHMWPE.

At the conclusion of the study, the cobalt chrome femoral components had linear scratching in an SI orientation, similar to that seen following wear simulation of TKRs.^44^ All the patellae had an increase in mean surface roughness. The Group 1 and Group 2 UHMWPE components had a polished region where there was a clear wear scar, but the evidence of pitting, scratching and delamination caused the elevated mean surface roughness.

## Conclusion

A method has been developed for experimental wear simulation of the PFJ under a malaligned condition, which resulted in a constant 5° external rotation applied to the patella button. For unworn patella buttons that were aged for 4 years in air, this malaligned condition did not influence wear after a 3 MC wear test compared to well-positioned implants and the geometry of the patella button did not influence wear rate. However, worn implants that were aged for 4 years in air and previously tested for 9 MC under standard gait conditions exhibited elevated wear rates, especially for round dome implants where subsurface cracking was visible. UHMWPE patella buttons aged in air for 8–10 years exhibited gross failure when tested under the malalignment condition, in some cases less than 1.5 MC. This shows that UHMWPE that has been gamma sterilised in an inert environment still has the potential for oxidative degradation when exposed to oxygen for extended durations.
